# Initial Validation of a Measure Assessing DSM-5-TR and ICD-11 Prolonged Grief in Children and Adolescents

**DOI:** 10.1007/s40653-026-00820-7

**Published:** 2026-02-16

**Authors:** Iris van Dijk, Paul A. Boelen, Jos de Keijser, Lonneke I. M. Lenferink

**Affiliations:** 1 Department of Clinical Psychology, Faculty of Social and Behavioural Sciences, Utrecht University, P.O. Box 80140, 3508 TC Utrecht, the Netherlands; 2 ARQ National Psychotrauma Centre, Nienoord 5, 1112 XE Diemen, the Netherlands; 3 Department of Clinical Psychology and Experimental Psychopathology, Faculty of Behavioral and Social Sciences, University of Groningen, Grote Kruisstraat 2/1, 9712 TS Groningen, the Netherlands; 4 Department of Psychology, Health & Technology, Faculty of Behavioural Management and Social Sciences, University of Twente, P.O. Box 217, 7500 AE Enschede, The Netherlands

**Keywords:** Prolonged grief disorder, DSM-5-TR, ICD-11, Screening, Children, Adolescents

## Abstract

Valid and reliable instruments to assess the most recent prolonged grief disorder (PGD) criteria, as defined in the DSM-5-TR and ICD-11, are lacking. This study aimed to provide an initial validation of the Traumatic Grief Inventory—Kids—Clinician-Administered (TGI-K-CA) for assessing Prolonged Grief Disorder (PGD) symptoms in bereaved children and adolescents according to DSM-5-TR and ICD-11 criteria. Ninety bereaved Dutch children (aged 8–18 years, *M*_*age*_ = 13.5, 61.1% female) participated. Bayesian confirmatory factor analyses and Bayesian correlational analyses were conducted to assess factor structure, temporal stability, and convergent and incremental validity. Prevalence rates and optimal cut-off scores of PGD caseness were determined. Findings regarding the psychometric properties were mixed. Neither a one-factor nor a two-factor model accurately represented the DSM-5-TR or ICD-11 PGD symptoms. Items related to disbelief and avoidance showed low factor loadings. Temporal stability over three months was weak. The TGI-K-CA showed convergent validity with other measures of depression, PTSD, quality of life and functional impairment. Prevalence rates for DSM-5-TR and ICD-11 PGD were both 7.8%. Optimal cut-off points for probable DSM-5-TR and ICD-11 PGD were ≥ 45 and ≥ 53 on the TGI-K-CA, respectively. Despite limitations such as small sample size and convenience sampling leading to possible biases, the TGI-K-CA appears to capture aspects of PGD symptoms in children. The TGI-K-CA facilitates research on screening tools for PGD and on DSM-5-TR and ICD-11 PGD criteria in children. Future studies should be conducted in larger and more diverse samples.

**Supplementary Information:**

The online version contains supplementary material available at https://doi.org/10.1007/s40653-026-00820-7.

Recently, prolonged grief disorder (PGD) has been included in the text revision of the fifth edition of Diagnostic and Statistical Manual for Psychiatric Disorders (DSM-5-TR; American Psychiatric Association, 2022) and the International Classification of Diseases (ICD-11; World Health Organization, 2018). PGD involves intense and persistent grief reactions that severely impair daily functioning. Although the inclusion of PGD has been widely seen as a clinical advancement, it has also raised concerns, particularly about the potential for pathologizing grief reactions (Cacciatore & Frances, 2022). However, failing to identify people – including children and adolescents^1^ – who are at risk of PGD may pose even greater risks. In children, PGD symptoms have been positively associated with adverse outcomes (e.g., aversive views of self, suicidality, and overall functional impairment; Melhem et al., 2007; Sandler et al., 2021, 2024). In addition, recognizing PGD in children is crucial in order to treat it effectively with first-choice treatments, including cognitive-behavioral therapy (Breen et al., 2023). Yet, studies examining PGD in children remain scarce, particularly in comparison to research on adult PGD. The formal recognition of PGD in DSM-5-TR and ICD-11 stresses the importance of research on PGD in children. As a first step, valid measures to assess PGD symptoms in children are necessary. The current study therefore evaluated the psychometric qualities of a screening tool for DSM-5-TR and ICD-11 PGD symptoms in children.

PGD, as defined in both the DSM-5-TR and ICD-11, can be classified in children after at least 6 months since the death of a loved one. PGD firstly involves separation distress. This refers to intense longing for, and preoccupation with the deceased person or the circumstances of their death. Secondly, a range of cognitive, emotional, and behavioral reactions (e.g., emotional numbness, identity disruption) characterizes PGD. Even though the DSM-5-TR and ICD-11 PGD criteria sets have similarities, there are notable differences. For instance, guilt, denial, and blame are included in the ICD-11, but not in the DSM-5-TR. Conversely, avoidance is included in the DSM-5-TR, but not in the ICD-11. See Table S1 for the symptoms of DSM-5-TR and ICD-11 PGD. To our knowledge, to date, one study investigated prevalence rates of ICD-11 PGD in children. It found that 12.4% of children met criteria for possible classification of PGD (Boelen et al., 2019).

Developing a measure to assess DSM-5-TR and ICD-11 PGD symptoms in children requires attention to two important considerations. First, although structured diagnostic interviews are the gold standard to classify disorders (but see Hyland & Shevlin, 2024), they are time-intensive and require training, making them less practical. Screening tools, on the other hand, provide a feasible and cost-effective way for identifying those at risk for PGD. When administered in an interview format by a clinician, such tools may further enhance response reliability, as clinicians can clarify items, probe inconsistencies, and encourage more reflective answers (Fresco et al., 2001). Although no formal classification can be made using a screening tool, it is a a valuable first step to identify who may be in need of further assessment and treatment.

Second, formulating items that align with the developmental level of children is crucial for reliable and valid assessment (Omrani et al., 2019). While research indicates that symptoms defining adult PGD also generally capture PGD among children at least as young as 7 years old (Boelen et al., 2019; Melhem et al., 2007, 2013), instruments designed for PGD are not necessarily suitable for children. That is, PGD may manifest differently across ages (Kaplow et al., 2012). The DSM-5-TR has therefore included several additions to symptoms that only apply to children. For example, avoidance of reminders that the deceased person is dead, may in children manifest as *efforts* to avoid (while in adults it may manifest as mere avoidance of reminders). Furthermore, due to children’s developing cognitive abilities, other adaptations to instruments, such as avoiding long items, are warranted to ensure accurate assessment (Omrani et al., 2019).

Existing screening instruments for prior criteria sets of maladaptive grief in children are the Inventory of Prolonged Grief for Children and for Adolescents (IPG-C and IPG-A; Spuij et al., 2012), the Traumatic Grief Inventory for Children (TGIC; Dyregrov et al., 2001), the Inventory for Complicated Grief-Revised for Children (ICG-RC; Melhem et al., 2013), and the Persistent Complex Bereavement Disorder (PCBD) Checklist (Kaplow et al., 2018). These instruments do not measure the latest DSM-5-TR and ICD-11 criteria of PGD. Instead, they measure now outdated maladaptive grief criteria.

To address the need of a brief, child-friendly measure of PGD in accordance with the DSM-5-TR and ICD-11 criteria, the Traumatic Grief Inventory – Kids – Clinician-Administered was developed for children of 8 to 18 years old (TGI-K-CA; Van Dijk et al., 2023). The TGI-K-CA stems from the Traumatic Grief Inventory – Self-Report Plus and Traumatic Grief Inventory – Clinician-Administered, developed for adults (TGI-SR + /CA; Lenferink et al., 2022, 2024). The development process of the TGI-K-CA involved input from five grief experts and seventeen bereaved children. Each item was evaluated by experts to ensure alignment with DSM-5-TR and ICD-11 PGD symptoms. Additionally, children of ages 8 through 17 years old provided feedback by verbalizing their thoughts while responding to the items. This approach facilitated identification of potential issues, such as children's difficulty with negatively phrased items, and allowed for adaptation of items. Furthermore, it provided confirmation that both younger and older children understood the items as intended (Van Dijk et al., 2023). This indicated that no separate versions needed to be constructed for children and adolescents, which was consistent with the PCBD Checklist that showed measurement invariance with respect to age (Hill et al., 2020).

In the current study, we evaluated the psychometric qualities of the TGI-K-CA in bereaved children. To do so, we evaluated the factor structure, the internal consistency, temporal stability, and incremental and convergent validity, and determined an optimal cut-off score for probable caseness of PGD in terms of DSM-5-TR and ICD-11. Based on earlier studies in bereaved adults that investigate the psychometric qualities of the TGI-SR + (Kokou-Kpolou et al., 2022; Lenferink et al., 2022, 2023a, b, 2024), we expected that a one-factor model would best represent the items that intend to measure DSM-5-TR and ICD-11 PGD symptoms. Moreover, we expected good internal consistency and temporal stability for DSM-5-TR and ICD-11 PGD items. With regard to the incremental validity, we anticipated that DSM-5-TR and ICD-11 PGD items relate to functional impairment above and beyond PTSD and depression. Furthermore, with respect to convergent validity, we expected that the DSM-5-TR and ICD-11 PGD items would be positively related to symptoms of PTSD, depression, and quality of life. Lastly, we also calculated rates of probable caseness and determined the optimal cut-off score.

## Methods

### Participants and Procedures

The current study was part of a larger project called ‘TrafVic Kids’. This project investigates the emotional consequences and treatment of psychological problems in children following the loss of a loved one due to a traffic accident. Ethical approval was obtained from Ethics Review Board of the Faculty of Social and Behavioural (21-0140). In the present study, participants, aged 8 through 18 years, had experienced the death (any cause) of family member or friend at least six months earlier and needed to have sufficient Dutch proficiency. Recruitment involved outreach through adults who participated in the [name]-project (a similar project in bereaved adults), social media advertisements, snowball sampling, outreach to mental health services, clinicians, (peer) support organizations, schools, and an online questionnaire accessible via a grief-related website. Given the focus of the [name] project on youth who lost close persons in fatal traffic accidents, recruitment efforts targeted traffic victim support organizations.

Participation involved of a 60-min online interview via Microsoft Teams. For youths that lost a loved one in a traffic accident, a follow-up measurement three months later was conducted. Active informed consent was obtained from children (of 12 years and older) and their parent(s) or caregiver(s) (when their child was 15 years or younger).

### Measures

#### Prolonged Grief Disorder Symptoms

PGD symptoms were assessed with the TGI-K-CA (Van Dijk et al., 2023). The 16-item TGI-K-CA is an interview administered by a clinician that can be used to screen for either DSM-5-TR or ICD-11 PGD. Twelve items refer to the eleven DSM-5-TR symptoms. Two items (i.e., “ In the past month, were you very sad because ____ has died?” and “In the past month, were you very angry because ____ has died?”) measure one symptom (i.e., DSM-5-TR C4, “Intense emotional pain”). The highest score on either of the two items is retained. Thirteen items refer to the thirteen ICD-11 symptoms. Children rated to what extent they experienced each symptom on 5-point Likert scales (1 = never, 5 = always). An example item is “In the past month, did you feel very alone because of the death of ____?”. Total scores range from 16 to 80. Sum scores for DSM-5-TR and ICD-11 PGD (excluding the functional impairment item) range from 10–50, and 12–60, respectively. Table 1 presents the mapping of items to DSM-5-TR and ICD-11 PGD symptoms.

**Table 1:** Item-mapping of TGI-K-CA items on DSM-5-TR and ICD-11 PGD symptoms

	Description of item	DSM-5-TR PGD	ICD-11 PGD
1	In the past month, did you miss ____ very much?	B1. Intense yearning/longing for the deceased person	B1. A persistent and pervasive longing for the deceased
2	In the past month, was your head full of thoughts and memories of (the circumstances surrounding the death of) _____?	B2. Preoccupation with thoughts or memories of the deceased person (in children and adolescents, preoccupation may focus on the circumstances of the death)	B2. A persistent and pervasive preoccupation with the deceased
3	In the past month, did you feel as if you lost a part of yourself?	C1. Identity disruption (e.g., feeling as though part of oneself has died) since the death	C7. Feeling one has lost a part of one’s self
4	In the past month, did you have trouble believing that ____ has died?	C2. Marked sense of disbelief about the death	C4. Denial
5	In the past month, did you try to stay away from everything that reminds you of _____, such as places, things, or thoughts?	C3. Avoidance of reminders that the person is dead (in children and adolescents, may be characterized by efforts to avoid reminders)	-
6	In the past month, were you very sad because ____ has died?	C4. Intense emotional pain (e.g., anger, bitterness, sorrow) related to the death	C1. Sadness
7	In the past month, were you very angry because ____ has died?	C4. Intense emotional pain (e.g., anger, bitterness, sorrow) related to the death	C3. Anger
8	In the past month, did you have trouble continuing with normal life (such as meeting your friends, or doing the things that you like)?	C5. Difficulty reintegrating into one’s relationships and activities after the death (e.g., problems engaging with friends, pursuing interests, or planning for the future)	C10. Difficulty in engaging with social or other activities
9	In the past month, did you have trouble having any feelings at all (such as sadness, anger, or happiness)?	C6. Emotional numbness (absence or marked reduction of emotional experience) as a result of the death	C9. Emotional numbness
10	In the past month, did you have the feeling that your life is empty and meaningless without _____?	C7. Feeling that life is meaningless as a result of the death	-
11	In the past month, did you feel very alone because of the death of ____?	C8. Intense loneliness as a result of the death	-
12	In the past month, did you feel guilty about the death of _____?	-	C2. Guilt
13	In the past month, did you blame other people that _____ is dead?	-	C5. Blame
14	In the past month, did you have trouble accepting that ____ is dead?	-	C6. Difficulty accepting the death
15	In the past month, did you have trouble feeling cheerful or happy?	-	C8. An inability to experience positive mood
16	In the past month, did you notice that you did less well in different areas (for example in school, or with friends and family)?	D. The disturbance causes clinically significant distress or impairment in social, occupational, or other important areas of functioning	E. The disturbance causes significant impairment in personal, family, social, educational, occupational or other important areas of functioning

DSM-5-TR = 5th text revised edition of the Diagnostic and Statistical Manual of Mental Disorders. ICD-11 = 11th edition of the International Classification of Diseases; PGD = Prolonged Grief Disorder

#### Depression Symptoms

The Patient Health Questionnaire-9 Modified for Teens (Aggarwal et al., 2017; PHQ-9 M) was used to measure depression severity. The PHQ-9 M consists of nine items that each reflect a DSM-5 symptom for major depressive disorder. The measure was translated from English to Dutch by forward–backward translation by two independent translators. Items refer to the past week (for example “Did you have little interest or pleasure in doing things?”). Responses are made on a 4-point Likert scale, ranging from 1 = not at all, to 4 = almost every day. Scores range from 9 to 36. The PHQ-9 M has adequate psychometric qualities (Nandakumar et al., 2019). In the current study, Cronbach’s alpha was .78.

#### Posttraumatic Stress Disorder Symptoms

The Child PTSD Symptom Scale for DSM-5 – Self Report (Foa et al., 2018; CPSS-5-SR) was used to assess symptom-levels of PTSD. The CPSS-5-SR consists of 27 items of which the first 20 items measure PTSD symptoms according to the DSM-5. The final seven items assess the impairment on daily functioning. In this study, only the first 20 items were used. The measure was translated from English to Dutch by forward–backward translation by two independent translators. Items refer to the past month. An example is “Having bad dreams or nightmares”. Responses are made on a 4-point Likert scale, ranging from 1 = not at all, to 5 = six or more times per week/severe). Scores range from 0 to 80. Research shows that the CPSS-5-SR has good psychometric qualities (Foa et al., 2018). In the current study, Cronbach’s alpha was .90.

#### Health-related Quality of Life

Health-related quality of life (HRQoL) was assessed by the KIDSCREEN-27 (Ravens-Sieberer et al., 2007). This 27-item questionnaire assesses five domains of HRQoL: physical well-being, mental well-being, autonomy and relationship with parent(s), social support and friends, and school. Items referred to the past week (for example “Have you had fun?”). Response categories ranged from 1 = poor to 5 = excellent, 1 = not at all to 5 = extremely, or from 1 = never to 5 = always, depending on the item. Scores range from 27 to 135. Higher scores indicate higher HRQoL, after four negatively formulated items are recoded. Research shows that the KIDSCREEN-27 is valid for children and adolescents of 8 – 18 years and that it has good test–retest reliability and internal consistency (Ravens-Sieberer et al., 2007). Cronbach’s alpha for the total scale in the current study was .90.

#### Demographic and Loss-related Variables

Demographic variables encompassed gender (male, female, or other), age, country of birth, and educational level. Additionally, loss-related variables included cause of death, time since loss and age of the deceased.

### Statistical Analyses

#### Factor Structure of Items Assessing DSM-5-TR and ICD-11 PGD Symptoms

First, for all items, descriptive statistics of the items were computed. This included the mean, standard deviation, mode, range, floor effect (the percentage of participants scoring 1 on the 5-point Likert-scale, using the commonly applied 15% floor effect threshold; Terwee et al., 2007), kurtosis and skewness. All items had kurtosis values of < 10 and skewness of < 3, which shows that item scores had univariate normal distributions (Kline, 2023). There were no missing values on the TGI-K-CA items.

Confirmatory factor analyses (CFAs) were conducted in *R* 4.2.3 (R Core Team, 2023) and RStudio 2023.03.0 + 386 (Posit Team, 2023). The CFAs were estimated with Bayesian estimation, which is expected to perform well under less than optimal data conditions such as small sample size and non-normal item distributions (Lee, 2007; Van De Schoot et al., 2017). The *blavaan* version 0.5–2 software package (Merkle et al., 2021) was used to perform Bayesian CFAs (BCFAs). For all items, scores were standardized to use in *blavaan*.

For both DSM-5-TR and ICD-11 PGD symptom items, a one-factor model was estimated first. Subsequently, a two-factor model was estimated, in which the two items of the B-criterion (regarding yearning/preoccupation with the deceased person) represented one factor and items of the C-criterion (regarding cognitive, emotional and behavioral reactions) represents a second factor. Bayesian analyses implement prior beliefs regarding the model parameters. Priors encompass a probability distribution representing the uncertainty about the parameters before observing the data. The priors for all factor loadings in the current study had a normal distribution with a mean of zero and a variance of ten (i.e., N(0,10)). These were the default, non-informative priors in *blavaan*. The software Stan, used within *blavaan*, employs the No-U-Turn-Sampler (NUTS) algorithm through Markov Chain Monte Carlo (MCMC) sampling. MCMC sampling is a technique to estimate the probability distribution of model parameters after incorporating the observed data (i.e., posterior distributions), by repeatedly generating random samples.

Model convergence was assessed using multiple indicators, that each should demonstrate satisfactory convergence. Indicators included: potential scale reduction factors (< 1.10 suggesting convergence), trace and autocorrelation plots, checking for local convergence by doubling iterations, calculating effective sample size (> 1000 suggesting convergence), and evaluating the histograms of posterior distributions for abnormalities (e.g., non-smoothness or gaps). In the end, convergence was achieved with priors estimated with 5000 iterations and posterior distributions with 2000 iterations.

Model fit was assessed by the following fit indices (Garnier-Villarreal & Jorgensen, 2020; Kline, 2023): the Bayesian Comparative Fit Index (BCFI) and the Bayesian Tucker-Lewis Index (BTLI) (both should be ≥ .90 for acceptable fit and ≥ .95 for excellent fit), and the Bayesian Root Mean Square Error of Approximation (BRMSEA; should be < .10 for acceptable fit and < .08 for excellent fit, but should be carefully interpreted because of lower reliability given the relatively small sample size; Kenny, 2020). Both models were not statistically compared using chi-square tests, given the relatively small sample size. Parsimony was considered in the model selection in case of similar model fit; the model with the least parameters was preferred over the more complex model. A sensitivity analysis on the priors was conducted to test the impact of different priors on the posterior distribution. This was done by using a normal prior of N(0,100) instead of the default N(0,10).

The WAMBS-checklist (‘*W*hen to worry and how to *A*void the *M*isuse of *B*ayesian *S*tatistics’) was followed throughout the BCFAs (Depaoli & Van De Schoot, 2017). This checklist describes main points that should be checked when conducting and reporting on Bayesian analyses. Readers are referred to Van De Schoot and Depaoli (2014) for an introduction to Bayesian methods.

#### Incremental Validity

A Bayesian multiple regression analysis was conducted in JASP version 0.16.2.0 (JASP Team, 2024). The score on the functional impairment item of the TGI-K-CA (item 16) was regressed on the total scores of either the DSM-5-TR or ICD-11 PGD total scores (excluding item 16 representing the functional impairment symptom), PTSD, and depression. Default priors were used for the parameters. The Bayes Factor (BF) was used to quantify the support for the alternative hypothesis (i.e., that there is a relationship between the two variables). For example, if BF = 10, there is ten times more support for the alternative hypothesis than for the null hypothesis. BFs are often presented in scientific notation due to their potentially large values, typically as *x* * 10^*x*^. For instance, a BF of 3,560,000 is expressed as BF = 3.56 * 10^6^. Furthermore, it was checked whether the confidence interval of the coefficient contained zero, which indicates the absence of a relationship.

#### Internal Consistency

The McDonald’s omega (ω) was computed to assess the internal consistency of both the DSM-5-TR and ICD-11 PGD symptom items (excluding the functional impairment item). Values of ω > 0.70 indicate an acceptable internal consistency (Hayes & Coutts, 2020). In addition, inter-item correlations were computed, with values > .80 indicating item redundancy (Diamantopoulos et al., 2012).

#### Temporal Stability

Bayesian correlational analyses were conducted in JASP. To account for the non-normally distributed data and the relatively small sample size, Kendalls tau (т) correlations were computed (Arndt et al., 1999). These included the summed items scores of the DSM-5-TR or the ICD-11 PGD symptoms at the first and second measurement point three months later (i.e., including only children who lost a loved one to a traffic accident). Default priors were used for the parameters. Values of т ≤ .20 were considered as weak, т > .20 ≤—.34 as moderate, and > .34 as strong (Gilpin, 1993). Additionally, the BF was examined, alongside the criterion that the entire confidence interval of т was > .20, indicative of moderate temporal stability.

#### Convergent Validity

Bayesian correlational analyses were performed in JASP. Kendalls tau correlations were computed to account for the non-normally distributed data and the relatively small sample size (Arndt et al., 1999). The correlations between either DSM-5-TR or ICD-11 PGD symptom total scores and symptoms of depression and PTSD, and HRQoL were assessed using default priors. Values of т were interpreted based on the confidence interval. The BF and whether the confidence interval of т contained zero were assessed.

#### Rates of Probable DSM-5-TR and ICD-11 PGD Caseness

Rates of probable caseness of DSM-5-TR PGD (i.e., the percentage of children meeting all criteria) were calculated by using the diagnostic scoring rule of the DSM-5-TR (APA, 2022). This includes the endorsement of at least one symptom of the B-criterion (items 1 and 2), at least three of eight symptoms of the C-criterion (items 3–11), and the D-criterion (item 16). Consistent with earlier studies (e.g., Lenferink et al., 2022), the endorsement of a symptom was defined as having a score of ≥ 4 on the corresponding item.

Probable caseness of ICD-11 PGD was determined by endorsement of at least one of the two symptoms of the B-criterion (items 1 and 2), at least one of the ten symptoms of the C-criterion (items 3, 4, 6–9, 12–15), and the D-criterion (item 16) (Mauro et al., 2019).

#### Determining Cut-off Scores for PGD Caseness

Using Receiver Operating Characteristic (ROC) analyses in SPSS, optimal cut-off scores to distinguish between people vs. not meeting the criteria for either DSM-5-TR or ICD-11 PGD disorder were determined. Consistent with research in adults (e.g., Lenferink et al., 2022, 2024), this was done for 1) the total TGI-K-CA score, 2) the summed score of DSM-5-TR PGD items (using the highest scored item of the two items representing symptom C4), and 3) the summed score of ICD-11 PGD items. ROC curves plotted the true-positive rate (i.e., sensitivity) against the false-positive rate (i.e., 1-specifity) for each possible cut-off score. The sensitivity and specificity rates were also calculated as percentages, after which a Youden’s index (i.e., sensitivity rates – (1-specificity rates)) can be determined. A value below 0.70 indicates poor accuracy for the distinction between (probable) caseness and non-caseness. Values between 0.70 and 0.80 are considered fair, between 0.80 and 0.90 are considered good, and between 0.90 and 1 are excellent (Ferraris, 2019).

### Open sciences Practices

The TGI-K-CA can be freely downloaded and is available in multiple languages from https://osf.io/2cmdp/. The current study was preregistered before completion of data collection (see https://osf.io/qwduk). Considering the smaller than anticipated sample size, a Bayesian instead of a frequentist approach for data-analyses was used.

## Results

### Sample Characteristics and Preliminary Analyses

Table 2 presents the demographic and loss-related characteristics and symptom-levels of PGD, PTSD, depression, and HrQoL within the sample. Ninety children participated in the study (*M*_*age*_ = 13.5 years, *SD* = 3.2). The majority of children were girls (61.1%), were born in the Netherlands (94.4%), and had lost a parent (50%), mostly due to physical illness (62.2%). The time since loss was on average 40 months (*SD* = 36.67) and ranged from 1.7 – 192.2 months.

**Table 2:** Demographic and loss-related characteristics, and symptom-levels of the sample (*N* = 90)

Demographic characteristics	
Gender (*n* (%))	
Girls	55 (61.1)
Boys	35 (38.9)
Other	0 (0.0)
Age (in years) (*M* (*SD*))	13.50 (3.23)
Country of birth (*n* (%))	
The Netherlands	85 (94.4)
Other	5 (5.6)
Loss-related characteristics	
Deceased is (*n* (%))	
Parent^a^	45 (50.0)
Grandparent	23 (25.6)
Sibling	10 (11.1)
Aunt/uncle	9 (10.0)
Other	3 (3.3)
Cause of death (*n* (%))	
Physical illness	56 (62.2)
Accident	28 (31.2)
Suicide	4 (4.4)
Other	2 (2.2)
Time since loss (in months) (*M* (*SD*))^b^	40.00 (36.67)
Symptom-levels, *M (SD)*; observed range	
DSM-5-TR PGD symptoms (TGI-K-CA)^c^	22.62 (7.76); 10–40
ICD-11 PGD symptoms (TGI-K-CA)^c^	26.43 (8.81); 12–48
PTSD symptoms (CPSS-5)	35.19 (12.49); 20–80
Depression symptoms (PHQ-9 M)	17.03 (4.81); 9–28
Health-related quality of life (KIDSCREEN-27)	105.79 (13.54); 65–131

DSM-5-TR = 5th text revised edition of the Diagnostic and Statistical Manual of Mental Disorders. ICD-11 = 11th edition of the International Classification of Diseases; PGD = Prolonged grief disorder; TGI-K-CA = Traumatic Grief Inventory – Kids – Clinician-Administered; PTSD = posttraumatic stress disorder; CPSS-5 = Child PTSD Symptom Scale for DSM-5; PHQ-9 M = Patient Health Questionnaire-9 Modified for Teens

^a^: Of whom two were stepparents

^b^: *N* = 73 due to missing parent-reported values, including uncompleted items or invalid dates

^c^: Excluding functional impairment item (item 16)

Descriptive analyses of item responses showed that mean scores ranged from 1.36 – 3.30. Floor effects ranged from 7.78% – 80.00%. These were present for fourteen out of sixteen items, and were highest for the items “Did you feel guilty about the death of _____?” and “Did you blame other people that _____ is dead?” (70.00% and 80.00% respectively; see Table S1 for descriptive statistics of all items).

### Factor Structure of Items Assessing DSM-5-TR PGD Symptoms

Fit indices of the one- and two-factor models of DSM-5-TR PGD are presented in Table 3. Both models did not show a reasonable fit, reflected by the high BRMSEA, and low BCFI and BTLI. The two-factor model had similar fit indices as the one-factor model. The standardized factor loadings of the one-factor model are presented in Table 4 and those of the two-factor model in Table S2. In both models, the items “Did you have trouble believing that ____ has died?” and “ Did you try to stay away from everything that reminds you of _____, such as places, things, or thoughts?” had low factor loadings (λ < 0.50). Given the similar fit indices, the most parsimonious one-factor model was retained for further analyses. Finally, the sensitivity analysis that changes the default priors, resulted in no meaningful changes.

**Table 3:** Fit indices of the Bayesian confirmatory factor analyses for DSM-5-TR and ICD-11 PGD items (*N* = 90)

	BCFI (90% CI)	BTLI (90% CI)	BRMSEA (90% CI)
DSM-5-TR PGD			
1-factor model	0.88 (0.86 – 0.91)	0.84 (0.81 – 0.88)	0.13 (0.11 – 0.14)
2-factor model	0.88 (0.86 – 0.91)	0.84 (0.80 – 0.87)	0.13 (0.11 – 0.14)
ICD-11 PGD			
1-factor model	0.79 (0.77 – 0.81)	0.72 (0.69 – 0.75)	0.15 (0.15 – 0.16)
2-factor model	0.79 (0.76 – 0.81)	0.72 (0.68 – 0.75)	0.16 (0.15 – 0.16)

*BCFI* Bayesian Comparative Fit Index; *CI* Confidence Interval; *DSM-5-TR* 5th text revised edition of the Diagnostic and Statistical Manual of Mental Disorders. ICD-11 = 11th edition of the International Classification of Diseases; *PGD *Prolonged grief disorder; *BTLI* Tucker Lewis Index; *BRMSEA* Bayesian Root Mean Square Error of Approximation

**Table 4:** Standardized factor loadings of the 1-factor models of DSM-5-TR and ICD-11 PGD (*N* = 90)

	Standardized factor loading
DSM-5-TR PGD	
Did you miss ____ very much? (B1)	0.821
Was your head full of thoughts and memories of (the circumstances surrounding the death of) _____? (B2)	0.757
Did you feel as if you lost a part of yourself? (C1)	0.614
Did you have trouble believing that ____ has died? (C2)	0.460
Did you try to stay away from everything that reminds you of _____, such as places, things, or thoughts? (C3)	0.360
Were you very sad because ____ has died? *or* In the past month, were you very angry because ____ has died? (C4)^a^	0.801
Did you have trouble continuing with normal life (such as meeting your friends, or doing the things that you like)? (C5)	0.685
Did you have trouble having any feelings at all (such as sadness, anger, or happiness)? (C6)	0.623
Did you have the feeling that your life is empty and meaningless without _____? (C7)	0.766
Did you feel very alone because of the death of ____? (C8)	0.749
ICD-11 PGD	
Did you miss ____ very much? (B1)	0.776
Was your head full of thoughts and memories of (the circumstances surrounding the death of) _____? (B2)	0.754
Were you very sad because ____ has died? (C1)	0.809
Did you feel guilty about the death of _____? (C2)	0.540
Were you very angry because ____ has died? (C3)	0.574
Did you have trouble believing that ____ has died? (C4)	0.480
Did you blame other people that _____ is dead? (C5)	0.251
Did you have trouble accepting that ____ is dead? (C6)	0.730
Did you feel as if you lost a part of yourself? (C7)	0.662
Did you have trouble feeling cheerful or happy? (C8)	0.691
Did you have trouble having any feelings at all (such as sadness, anger, or happiness)? (C9)	0.570
Did you have trouble continuing with normal life (such as meeting your friends, or doing the things that you like)? (C10)	0.691

DSM-5-TR = 5th text revised edition of the Diagnostic and Statistical Manual of Mental Disorders; ICD-11 = 11th edition of the International Classification of Diseases; PGD = Prolonged grief disorder

^a^ Symptom is measured by two items. The highest score on either of the two items is retained

### Factor Structure of Items Assessing ICD-11 PGD Symptoms

Table 3 shows the fit indices of the one- and two-factor models of ICD-11 PG. The BRMSEA, the BCFI and BTLI did not indicate an acceptable fit and were similar across both models. The standardized factor loadings of the one-factor model are presented in Table 4 and those of the two-factor model in Table S2. There were two items that had factor loadings in both models (λ < 0.50): “Did you have trouble believing that ____ has died?” and “Did you blame other people that _____ is dead?”. In addition, three items had moderate factor loadings (0.50 > λ < 0.60): “Did you feel guilty about the death of _____?”, “Were you very angry because ____ has died?”, “Did you have trouble having any feelings at all (such as sadness, anger, or happiness)?”. Given the similar fit indices, the most parsimonious one-factor model was retained for further analyses. The sensitivity analysis that changes the default priors, resulted in no meaningful changes.

### Incremental Validity

For DSM-5-TR PGD, when accounting for the shared variance between PGD, depression, and PTSD, PGD had a small relationship with functional impairment (see Table 5 for the results regarding incremental validity). There was no relationship between depression and functional impairment. Furthermore, there was no relationship between PTSD and functional impairment.

**Table 5:** Outcomes of multiple regression analysis and correlational analyses for incremental and convergent validity, and temporal stability

Correlate	Incremental validity^a^ (*b*, 95% CI, BF)					
	DSM-5-TR PGD	Depression (DSM-5-TR model)	PTSD (DSM-5-TR model)	ICD-11 PGD	Depression (ICD-11 model)	PTSD (ICD-11 model)
Functional impairment	*b* = 0.07, 95% CI: 0.03—0.10, BF = 107.52	*b* = 0.03, 95% CI: -0.01—0.08, BF = 1.56	*b* = 0.02, 95% CI: < -0.01—0.04, BF = 1.85	*b* = 0.06, 95% CI: 0.03—0.09, BF = 376.80	*b* = 0.03, 95% CI: < 0.01—0.09, BF = 2.06	*b* = 0.01, 95% CI: < -0.01—0.04, BF = 1.35
	Convergent validity (т, 95% CI, BF)					
	DSM-5-TR PGD			ICD-11 PGD		
Depression	т = 0.40, 95% CI: 0.25 – 0.52, BF = 482970.51			т = 0.37, 95% CI: 0.22 – 0.50; BF = 90607.15		
PTSD	т = 0.48, 95% CI: 0.33 – 0.60; BF = 6.76 * 10^8^			т = 0.55, 95% CI: 0.40 – 0.67; BF = 7.77 * 10^11^		
Quality of life	т = -0.35, 95% CI: -0.47 – -0.20; BF = 16532.38			т = -0.33, 95% CI: -0.46 – -0.19; BF = 6185.66		
	Temporal stability (т, 95% CI, BF)					
	DSM-5-TR PGD W1			ICD-11 PGD W1		
DSM-5-TR PGD W2	т = 0.31, 95% CI: -0.06—0.57, BF = 1.14			-		
ICD-11 PGD W2	-			т = 0.36, 95% CI: -0.02—0.61, BF = 1.80		

^a^ Accounted for shared variance between DSM-5-TR/ICD-11 PGD, PTSD, and depression

As shown in Table 5, similar results were found when ICD-11 PGD was included instead of DSM-5-TR PGD. PGD had a small relationship with functional impairment. There was no relationship between depression and functional impairment. Moreover, there was no relationship between PTSD and functional impairment.

### Internal Consistency

For DSM-5-TR PGD items, McDonald’s omega was .88. For ICD-11 PGD items, McDonald’s omega was .89. The inter-item correlations for all items are presented in Table S2. Inter-item correlations ranged between .17 and .79 for the DSM-5-TR PGD items, and between .03 and .79 for ICD-11 PGD items. This does not indicate item redundancy.

### Convergent Validity

As shown in Table 5, DSM-5-TR PGD had a positive moderate to strong association with depression symptoms, a positive strong association with symptoms of PTSD, and a negative moderate to strong association with HRQoL.

A similar positive moderate to strong association was found for ICD-11 PGD and depression symptoms. Furthermore, ICD-11 PGD had a positive strong association with PTSD symptoms and a negative moderate to strong association with HRQoL.

### Temporal Stability

Comparing the DSM-5-TR and ICD-11 PGD criteria sets at the first and second measurement point (*N* = 16 children, *M* = 3.4 months between first and measurement point, *SD* = 0.35), the BF that was close to 1 indicated weak evidence for temporal stability for both criteria sets (see Table 5).

### Rates of Probable DSM-5-TR and ICD-11 PGD Caseness

Seven children (7.8%) met criteria for probable DSM-5-TR PGD. For ICD-11 PGD, also seven children (7.8%) met the criteria. Subsequent analysis showed that both criteria sets led to the same children being classified with PGD, indicating 100% diagnostic agreement.

### Determing Cut-off Scores for PGD Caseness

When the total TGI-K-CA score (16 items; possible range is 16–80) was used, the optimal cut-off for probable caseness of DSM-5-TR PGD was ≥ 45 (AUC = 0.95, 95% CI: 0.90 – 1.00). Using this cut-off score, 100% of cases were correctly identified as DSM-5-TR PGD, and 12.0% was incorrectly identified. Youden’s index was good (*J* = 0.88).

The optimal cut-off score was ≥ 53 for ICD-11 PGD (AUC = 0.95, 95% CI: 0.90 – 1.00), with 100% of cases being correctly identified and 10.4% being incorrectly identified (*J* = 0.88; this is good).

The optimal cut-off for probable caseness of DSM-5-TR PGD when only including the ten DSM-5-TR PGD symptoms (possible range is 10–50) was ≥ 29 (AUC = 0.94, 95% CI: 0.88 – 0.99). Using this cut-off score, 100% of probable cases were correctly identified as PGD, and 16.9% was incorrectly identified. Youden’s index was good (*J* = 0.83).

The optimal cut-off score for probable caseness of ICD-11 PGD when only including the twelve ICD-11 PGD symptoms (possible range is 12–60) was ≥ 33 (AUC = 0.92, 95% CI: .86 – 0.99). This score yielded 100% correctly identified probable cases, and 18% incorrectly identified cases (*J* = 0.82; this is good).

We repeated all the analyses, excluding three children whose loved one died less than 6 months earlier. The results did not change meaningfully (findings are not reported here, but can be requested from the corresponding author).

## Discussion

The current study provided an initial validation of the TGI-K-CA, an interview-based screening tool assessing DSM-5-TR and ICD-11 PGD symptoms, in 90 bereaved children. The results present a mixed picture of the six studied indicators of psychometric quality. Firstly, neither a one-factor nor a two-factor model accurately represented the items measuring DSM-5-TR or ICD-11 PGD symptoms. This finding was contrary to the expectation of a one-factor model, based on literature on the TGI-SR + /CA for bereaved adults (Kokou-Kpolou et al., 2022; Lenferink et al., 2022, 2023a, b, 2024). The suboptimal model fit may be partly attributed to floor effects, observed across almost all items. Floor effects, characterized by a high proportion of responses in the lowest response category, likely contributed to low factor loadings by limiting response variability, which is required to accurately differentiate between items.

While floor effects may contribute to low factor loadings and poor overall model fit, the poor performance of specific items within the DSM-5-TR and ICD-11 PGD criteria sets may extend beyond our sample. For instance, in our study, the items related to disbelief in both criteria sets and avoidance in DSM-5-TR consistently showed low factor loadings. These findings are consistent with results from the IPG-C/A, which also showed low factor loadings for these items among children (Spuij et al., 2012). Additionally, studies on the TGI-SR + /CA in adults have also identified relatively low factor loadings for the item related to avoidance (Lenferink et al., 2024). This pattern suggests potential conceptual inconsistencies inherent to the DSM-5-TR and ICD-11 criteria sets themselves. For example, whereas the avoidance item measures a behavioral symptom, many other symptoms are emotional or cognitive in nature, which could complicate its integration into a cohesive factor with the other symptoms within the criteria sets. The low factor loadings of the avoidance item in our study as well in others may also stem from the difficulty of assessing avoidance via self-report, a concern raised beyond research on PGD (cf. Gamez et al., 2010).

Furthermore, age-related manifestations of symptoms that may not fully captured by the DSM-5-TR and ICD-11 criteria sets may have influenced our findings. While children in our study understood the TGI-K-CA items as intended during cognitive interviews (Van Dijk et al., 2023), there may be discrepancies between on the one hand the DSM-5-TR and ICD-11 criteria and on the other hand how PGD symptoms manifest during childhood and adolescence. Even though the current study aimed to closely align with the DSM-5-TR and ICD-11 PGD symptoms, this suggests a potential need for a more bottom-up approach in defining PGD criteria sets for children. Integrating qualitative and quantitative data to assess age-related PGD manifestations emphasizes a developmental approach to PGD, as advocated by other researchers (e.g., Alvis et al., 2023; Andriessen et al., 2018; Kaplow et al., 2012; Lytje & Dyregrov, 2024). For instance, future research could apply this by first collecting qualitative data from children’s own descriptions of intense, disabling grief to develop an elaborate item pool. Data-driven methods could then be used to refine and validate the item pool.

Secondly, although there was a moderate to strong positive relationship between PGD symptoms at the first and second time point three months later, the Bayesian analyses indicated weak evidence for temporal stability. One explanation could be the small sample size, which may have limited the statistical power to detect stable relationships over time. Additionally, PGD symptoms can naturally fluctuate over time as found in bereaved adults (Lenferink et al., 2023a, b). The instrument might have captured these natural fluctuations. Future research should further explore the temporal stability within different, including briefer, time frames.

Thirdly, initial evidence for convergent validity was provided by moderate to strong positive correlations between DSM-5-TR and ICD-11 PGD symptoms and symptoms of depression and PTSD. This aligns with associations found in prior research between PGD symptoms and symptoms of depression and PTSD in children (e.g., Melhem et al., 2007; Spuij et al., 2012). Additionally, the moderate to strong negative correlation with HRQoL provided further support for convergent validity. This underscores the interconnected yet distinct nature of mental illness and positive mental health (Keyes, 2005).

Fourthly, when accounting for the shared variance between symptoms of PGD, depression, and PTSD, symptoms of PGD had a small relationship with functional impairment. This provides initial evidence for the incremental validity of the TGI-K-CA. These findings align with prior studies among both children and adults, that PGD symptoms explain functional impairment beyond symptoms of depression and PTSD (Melhem et al., 2007; Spuij et al., 2012).

Fifthly, the estimated prevalence rates for both DSM-5-TR PGD and ICD-11 PGD were 7.8%. This is lower than the prevalence rates reported in prior research on youth PGD of 10.4 to 32% (Falala et al., 2024). The 100% diagnostic agreement rate between DSM-5-TR and ICD-11 criteria sets contrasts with lower agreement rates of 40% for PCBD and ICD-11 PGD in children and 51% for DSM-5-TR and ICD-11 PGD in adults (Boelen et al., 2019; Haneveld et al., 2022). The high diagnostic agreement supports the use of the instrument to detect both DSM-5-TR and ICD-11 PGD. However, given the limited generalizability of past findings using prior criteria sets and measures, and due to variations in study populations and types of loss examined, caution is warranted when comparing these rates between studies.

Sixthly, optimal cut-off points for probable DSM-5-TR and ICD-11 PGD were determined at ≥ 45 and ≥ 53 on the total TGI-K-CA score, respectively. When considering DSM-5-TR symptoms only, the optimal cut-off was ≥ 29, and for ICD-11 symptoms, the cut-off was ≥ 33. These cut-offs showed good sensitivity and specificity. However, the high percentage of correctly identified cases may reflect the low prevalence of PGD caseness in the sample. This may affect the precision of the sensitivity and specificity estimates. Therefore, the cut-off points should be cautiously used. Future research should examine if thresholds differ between relevant subgroups of bereaved children and youngsters.

There are several limitations that should be noted. First, the relatively small sample size precluded classical frequentist statistical analyses. Nevertheless, Bayesian statistics provide a valid alternative, as it may be more accurate than frequentist statistics in the case of limited data, reducing the risk of false positives (Natesan, 2015; Van De Schoot et al., 2017). Bayesian statistics are increasingly used in evaluations of psychometric properties of instruments that include relatively small sample sizes (e.g., Golay et al., 2013). Second, convenience sampling was used. This may have resulted in self-selected biases, whereby children and their consenting parents with fewer symptoms may have been more likely to participate. The observed floor effects in our study could therefore reflect a bias towards a well-functioning sample. On a similar note, the sample was relatively homogeneous regarding gender, country of birth, who the deceased person was, and which type of loss occurred. Most children were girls, were born in the Netherlands, had lost a parent, and had lost their loved one to a physical illness. Thus, generalization of the findings to other groups (e.g., bereaved children due to suicide) should be done cautiously. Nonetheless, given the limited range of PGD symptoms in our sample, associations between DSM-5-TR and ICD-11 PGD with related constructs may have been attenuated. These associations could be more pronounced in more diverse or symptomatic populations. Future research with larger and more heterogeneous samples is therefore important to further evaluate the psychometric qualities of the TGI-K-CA, including the use of more advanced psychometric approaches that require larger sample sizes.

Despite these limitations, the findings from this study still offer important insights. While the TGI-K-CA items cannot be represented by a unidimensional factor, they still seem to capture aspects of the construct, as reflected in their associations with related constructs. Importantly, the TGI-K-CA is intended as a screening instrument, as a first step to decide whether further research is needed into the classification of PGD. To fully understand grief in children and adolescents, it is essential to consider their broader social environment (Alvis et al., 2023; Andriessen et al., 2020). Therefore, the TGI-K-CA should ideally be used as part of a systemic assessment, incorporating perspectives from caregivers, teachers, or other informants. While the parent-report version of the TGI-K-CA has yet to be validated, other instruments such as the caregiver-report version of the International Grief Questionnaire (IGQ-CG; Redican et al., 2024) may offer complementary information. At this stage, the TGI-K-CA allows for further research on screening tools for symptoms of PGD in children, and on the DSM-5-TR and ICD-11 criteria sets. Future studies should be conducted in larger and more diverse samples.

## Supplementary Information

Below is the link to the electronic supplementary material.

Supplementary file1 (DOCX 29 KB)

## Data Availability

The data that support the findings of this study are available from the corresponding author upon reasonable request.
