# Supplementary material for: Initial Validation of a Measure Assessing DSM-5-TR and ICD-11 Prolonged Grief in Children and Adolescents
Source: J Child Adolesc Trauma. 2026 Feb 16;19(3):1111–23. doi: 10.1007/s40653-026-00820-7 (PMC13546224; doi:10.1007/s40653-026-00820-7)
Supplement: Supplementary file 1 [file 40653_2026_820_MOESM1_ESM.docx]

**Table S1**

|  | Description of item | Mean | SD | Mode | Range | Floor effect (%) | Skewness | Kurtosis |
| --- | --- | --- | --- | --- | --- | --- | --- | --- |
| 1 | In the past month, did you miss ____ very much? | 3.30 | 1.15 | 3 | 1-5 | 7.78 | -0.25 | -0.57 |
| 2 | In the past month, was your head full of thoughts and memories of (the circumstances surrounding the death of) _____? | 2.92 | 1.01 | 3 | 1-5 | 11.11 | -0.18 | -0.05 |
| 3 | In the past month, did you feel as if you lost a part of yourself? | 2.13 | 1.24 | 1 | 1-5 | 43.33 | 0.72 | -0.77 |
| 4 | In the past month, did you have trouble believing that ____ has died? | 2.26 | 1.25 | 1 | 1-5 | 41.11 | 0.45 | -1.01 |
| 5 | In the past month, did you try to stay away from everything that reminds you of _____, such as places, things, or thoughts? | 1.61 | 1.01 | 1 | 1-5 | 67.78 | 1.92 | 2.79 |
| 6 | In the past month, were you very sad because ____ has died? | 2.70 | 1.11 | 3 | 1-5 | 15.56 | 0.11 | -0.78 |
| 7 | In the past month, were you very angry because ____ has died? | 1.91 | 1.02 | 1 | 1-4 | 46.67 | 0.82 | -0.25 |
| 8 | In the past month, did you have trouble continuing with normal life (such as meeting your friends, or doing the things that you like)? | 1.83 | 1.05 | 1 | 1-4 | 54.44 | 0.88 | -0.64 |
| 9 | In the past month, did you have trouble having any feelings at all (such as sadness, anger, or happiness)? | 2.04 | 1.08 | 1 | 1-4 | 43.33 | 0.51 | -1.12 |
| 10 | In the past month, did you have the feeling that your life is empty and meaningless without _____? | 1.77 | 1.92 | 1 | 1-4 | 56.67 | 1.02 | -0.31 |
| 11 | In the past month, did you feel very alone because of the death of ____? | 1.92 | 1.07 | 1 | 1-5 | 46.67 | 1.00 | 0.17 |
| 12 | In the past month, did you feel guilty about the death of _____? | 1.52 | 1.58 | 1 | 1-4 | 70.00 | 1.58 | 1.28 |
| 13 | In the past month, did you blame other people that _____ is dead? | 1.36 | 0.83 | 1 | 1-5 | 80.00 | 2.56 | 6.28 |
| 14 | In the past month, did you have trouble accepting that ____ is dead? | 2.37 | 1.24 | 1 | 1-5 | 33.33 | 0.46 | -0.86 |
| 15 | In the past month, did you have trouble feeling cheerful or happy? | 2.08 | 1.10 | 1 | 1-5 | 36.67 | 0.92 | 0.10 |
| 16 | In the past month, did you notice that you did less well in different areas (for example in school, or with friends and family)? | 1.84 | 1.14 | 1 | 1-5 | 54.44 | 1.29 | 0.82 |

**Table S2**

Standardized factor loadings of the 2-factor models of DSM-5-TR and ICD-11 PGD (*N* = 90)

|  | Standardized factor loading |
| --- | --- |
| DSM-5-TR PGD |  |
| Did you miss ____ very much? (B1) | 0.818 |
| Was your head full of thoughts and memories of (the circumstances surrounding the death of) _____? (B2) | 0.746 |
| Did you feel as if you lost a part of yourself? (C1) | 0.634 |
| Did you have trouble believing that ____ has died? (C2) | 0.460 |
| Did you try to stay away from everything that reminds you of _____, such as places, things, or thoughts? (C3) | 0.360 |
| Were you very sad because ____ has died? *or* In the past month, were you very angry because ____ has died? (C4)^a^ | 0.801 |
| Did you have trouble continuing with normal life (such as meeting your friends, or doing the things that you like)? (C5) | 0.685 |
| Did you have trouble having any feelings at all (such as sadness, anger, or happiness)? (C6) | 0.623 |
| Did you have the feeling that your life is empty and meaningless without _____? (C7) | 0.766 |
| Did you feel very alone because of the death of ____? (C8) | 0.749 |
| ICD-11 PGD |  |
| Did you miss ____ very much? (B1) | 0.814 |
| Was your head full of thoughts and memories of (the circumstances surrounding the death of) _____? (B2) | 0.756 |
| Were you very sad because ____ has died? (C1) | 0.764 |
| Did you feel guilty about the death of _____? (C2) | 0.550 |
| Were you very angry because ____ has died? (C3) | 0.580 |
| Did you have trouble believing that ____ has died? (C4) | 0.475 |
| Did you blame other people that _____ is dead? (C5) | 0.252 |
| Did you have trouble accepting that ____ is dead? (C6) | 0.722 |
| Did you feel as if you lost a part of yourself? (C7) | 0.666 |
| Did you have trouble feeling cheerful or happy? (C8) | 0.702 |
| Did you have trouble having any feelings at all (such as sadness, anger, or happiness)? (C9) | 0.578 |
| Did you have trouble continuing with normal life (such as meeting your friends, or doing the things that you like)? (C10) | 0.697 |

*Note.* DSM-5-TR = 5th text revised edition of the Diagnostic and Statistical Manual of Mental Disorders; ICD-11 = 11th edition of the International Classification of Diseases; PGD = Prolonged grief disorder.

^a^ Symptom is measured by two items. The highest score on either of the two items is retained.

**Table S3**

Inter-item correlations for items representing DSM-5-TR and ICD-11 PGD symptoms (*N* = 90)

|  | 1 | 2 | 3 | 4 | 5 | 6 | 7 | 8 | 9 | 10 | 11 | 12 |
| --- | --- | --- | --- | --- | --- | --- | --- | --- | --- | --- | --- | --- |
| DSM-5-TR PGD symptom |  |  |  |  |  |  |  |  |  |  |  |  |
| 1. B1 | 1 |  |  |  |  |  |  |  |  |  |  |  |
| 2. B2 | .594 | 1 |  |  |  |  |  |  |  |  |  |  |
| 3. C1 | .534 | .477 | 1 |  |  |  |  |  |  |  |  |  |
| 4. C2 | .372 | .432 | .301 | 1 |  |  |  |  |  |  |  |  |
| 5. C3 | .183 | .348 | .286 | .214 | 1 |  |  |  |  |  |  |  |
| 6. C4 | .788 | .676 | .488 | .393 | .172 | 1 |  |  |  |  |  |  |
| 7. C5 | .499 | .528 | .535 | .356 | .206 | .491 | 1 |  |  |  |  |  |
| 8. C6 | .353 | .454 | .473 | .166 | .209 | .412 | .457 | 1 |  |  |  |  |
| 9. C7 | .591 | .486 | .560 | .207 | .390 | .536 | .488 | .618 | 1 |  |  |  |
| 10. C8 | .576 | .444 | .516 | .364 | .284 | .536 | .530 | .515 | .637 | 1 |  |  |
| ICD-11 PGD symptom |  |  |  |  |  |  |  |  |  |  |  |  |
| 1. B1 | 1 |  |  |  |  |  |  |  |  |  |  |  |
| 2. B2 | .594 | 1 |  |  |  |  |  |  |  |  |  |  |
| 3. C1 | .791 | .652 | 1 |  |  |  |  |  |  |  |  |  |
| 4. C2 | .292 | .391 | .265 | 1 |  |  |  |  |  |  |  |  |
| 5. C3 | .457 | .368 | .379 | .442 | 1 |  |  |  |  |  |  |  |
| 6. C4 | .372 | .432 | .349 | .321 | .235 | 1 |  |  |  |  |  |  |
| 7. C5 | .162 | .295 | .122 | .259 | .379 | .138 | 1 |  |  |  |  |  |
| 8. C6 | .644 | .571 | .646 | .385 | .529 | .507 | .290 | 1 |  |  |  |  |
| 9. C7 | .534 | .477 | .530 | .319 | .319 | .301 | .060 | .394 | 1 |  |  |  |
| 10. C8 | .523 | .409 | .516 | .512 | .461 | .154 | .077 | .390 | .527 | 1 |  |  |
| 11. C9 | .353 | .454 | .364 | .383 | .368 | .166 | .193 | .283 | .473 | .645 | 1 |  |
| 12. C10 | .499 | .528 | .495 | .590 | .337 | .356 | .031 | .374 | .535 | .611 | .457 | 1 |
